# Exact Tile-Based Segmentation Inference for Images Larger than GPU Memory

**DOI:** 10.6028/jres.126.009

**Published:** 2021-06-03

**Authors:** Michael Possolo, Peter Bajcsy

**Affiliations:** 1 National Institute of Standards and Technology, Gaithersburg, MD 20899, USA

**Keywords:** artificial intelligence, convolutional neural networks, effective receptive field, out-of-core processing, semantic segmentation

## Abstract

We address the problem of performing exact (tiling-error free) out-of-core semantic segmentation inference of arbitrarily large images using fully convolutional neural networks (FCN). FCN models have the property that once a model is trained, it can be applied on arbitrarily sized images, although it is still constrained by the available GPU memory. This work is motivated by overcoming the GPU memory size constraint without numerically impacting the final result. Our approach is to select a tile size that will fit into GPU memory with a halo border of half the network receptive field. Next, stride across the image by that tile size without the halo. The input tile halos will overlap, while the output tiles join exactly at the seams. Such an approach enables inference to be performed on whole slide microscopy images, such as those generated by a slide scanner. The novelty of this work is in documenting the formulas for determining tile size and stride and then validating them on U-Net and FC-DenseNet architectures. In addition, we quantify the errors due to tiling configurations which do not satisfy the constraints, and we explore the use of architecture effective receptive fields to estimate the tiling parameters.

## 1 Introduction

The task of semantic segmentation, i.e., assigning a label to each image pixel, is often performed using deep learning based convolutional neural networks (CNNs) [1, 2]. A subtype of CNN that only uses convolutional layers is called a "fully convolutional neural network" (FCN), which can be used with input of arbitrary size. Both U-Net ^1^1Certain commercial equipment, instruments, or materials are identified in this paper to foster understanding. Such identification does not imply recommendation or endorsement by the National Institute of Standards and Technology, nor does it imply that the materials or equipment identified are necessarily the best available for the purpose.[2] and the original FCN network [3] are examples of FCN type CNNs. FCNs enable the network to be trained on images much smaller than those of interest at inference time as long as the resolution is comparable. For example, one can train a U-Net model on (512 × 512) pixel tiles and then perform graphics processing unit (GPU) based inference on arbitrarily sized images, provided the GPU memory can accommodate the model coefficients, network activations, application code, and an input image tile. This decoupling of the training and inference image sizes means that semantic segmentation models can be applied to images that are much larger than the memory available on current GPUs.

Images larger than GPU memory can also be used to train the model if they are first broken into tiles. In fact, that is a fairly common practice when working with large-format images. Interestingly, the same edge effects which cause tiling errors during inference also affects the training process. Therefore, the tiling methodology presented here could also be used to generate tiles for network training.

The ability of FCN networks to perform inference on arbitrarily large images differs from other types of CNNs where the training and inference image sizes must be identical. Usually, this static image size requirement is not a problem since the input image size is expected to be static, or images can be resized within reason to ft the network. For example, if one trained a CNN on ImageNet [4] to classify pictures into two classes: {Cat, Dog}, then the content of the image does not change drastically if the cat photo is resized to (224 × 224) pixels before inference, provided the resolution is not altered considerably. Convolutional networks are not yet capable of strong generalization across scales [5, 6], so the inference time pixel resolution needs to approximately match the training time resolution or accuracy can suffer.

In contrast, there are applications where resizing the image is not acceptable due to loss of information. For example, in digital pathology, one cannot take a whole slide microscopy image generated by a slide scanner (upwards of 10 gigapixels) and ft it into GPU memory; nor can one reasonably resize the image because too much image detail would be lost.

Our work is motivated by the need to design a methodology for performing inference on arbitrarily large images on GPU memory-constrained hardware in those applications where the loss of information due to image resizing is not acceptable. This method can be summarized as follows. The image is broken down into non-overlapping tiles. The local context of each tile is defined by the halo border (ghost region), which is included only when performing inference. The tile size should be selected so that when the halo border is included the whole image will ft into GPU memory. The network receptive field [7] is the set of all input pixels which can influence a given output pixel. We use the receptive field to define the halo border as half the network receptive field to ensure that pixels on the edge of the non-overlapping tiles have all of the required local context as if computed in a single forward pass.

There are three important concepts required for this tile-based (out-of-core) processing scheme.

1. Zone of Responsibility (ZoR): a rectangular region of the output image currently being computed, a.k.a. the output tile size.
2. Halo: minimum horizontal and vertical border around the ZoR indicating the local context that the FCN requires to accurately compute all pixels within the ZoR. This value is equal to half of the receptive field size of the model architecture being used. This is derived from the definition of the convolution network, each output pixel in the ZoR is dependent upon other pixels in either the ZoR or in the halo.
3. Stride: the stride (in pixels) across the source image used to create tiles. The stride is fixed at the ZoR size.

The original U-Net paper [2] briefly hinted at the feasibility of an inference scheme similar to the one we present in this paper, but it did not fully quantify and explain the inference mechanism. The novelty of our work lies in presenting a methodology for tiling-error-free inference over images that are larger than available GPU memory for processed data.

## 2 Related Work

Out-of-core, tile-based processing is a common approach in the high performance computing (HPC) field where any local signal processing filter can be applied to carefully decomposed subregions of a larger problem [8]. The goal is often computation acceleration via task or data parallelization. These tile-based processes for the purpose of task parallelization also reduce the active working memory required at any given point in the computation.

It has been known since the initial introduction of FCN models that they can be applied via shift-and-stitch methods as if the FCN were a single filter [3, 9]. The original U-Net paper [2] also hinted at inference performed on arbitrary sized images in its Figure 2. However, none of the past papers mentioning shift-and-stitch discuss the methodology for performing out-of-core inference on arbitrarily sized images.

There are two common approaches for applying CNN models to large images: sliding window (overlapping tiles) and patch-based. Sliding windows (*i.e.*, overlapping tiles) have been used for object detection [10, 11] and for semantic segmentation [12, 13]. Patch-based inference also supports arbitrarily large images, but it can be very inefficient [13, 14].

Huang *et al.* [15] and Iglovikov *et al.* [16] both proposed sliding window approaches. Huang *et al.* [15] directly examined the problem of operating on images for which inference cannot be performed in a single forward pass. The authors focused on different methods for reducing but not eliminating the error in labeling that arises from different overlapping tile-based processing schemes. They examined label averaging and the impacts of different tile sizes on the resulting output error and concluded that using as large a tile as possible will minimize the error. Huang *et al.* [15] also examined the effects of zero-padding, documenting the error it introduces. At no point did they produce tiling-error free inference. Iglovikov *et al.* [16] remarked upon the error in the output logits at the tile edges during inference and suggested overlapping predictions or cropping the output to reduce that error.

Patch based methods for dealing with large images predict a central patch given a larger local context. Mnih [17] predicted a (16 × 16) pixel patch from a (64 × 64) pixel area for road segmentation from aerial images. This approach will scale to arbitrarily large images, and if a model architecture with a receptive field of less than 48 pixels is used, then it will have zero error from the tiling process. Saito *et al.* [18] used a similar patch-based formulation to Mnih, predicting a center patch given a large local context; however, they added a model averaging component by predicting eight slightly offset versions of the same patch and then combining the predictions.

Our ability to perform error-free tile-based inference relies on the limited receptive field of convolutional layers. Luo *et al.* [19] discussed the receptive fields of convolutional architectures, highlighting the fact that for a given output pixel, there is limited context/information from the input that can influence that output [7]. Input data outside the receptive field cannot influence that output pixel [19]. The receptive field of a model is highly dependent upon architecture and layer connectivity. We use the theoretical underpinning of receptive fields to identify the size of an image tile sufficient for performing inference without tiling-based errors.

To the best of our knowledge, no published method fully explores a methodology for performing error-free tile-based (out-of-core) inference of arbitrarily large images. While tile-based processing schemes have been outlined, the past publications do not provide a framework for achieving error-free tile-based inference results. Our approach does not handle layers with dynamic inference or variable receptive fields like stand-alone self-attention [20], squeeze and excitation layers [21], deformable convolutions [22], or non-local layers [23].

## 3 Methods

**Fig. 1:**
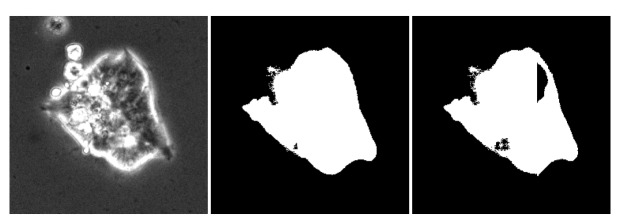
(Left) A (250 × 250) pixel subregion of a grayscale stem cell colony image being segmented by U-Net. (Center) Segmentation result with proper halo border of 96 pixels. (Right) Segmentation result with artifacts due to tiling (halo border is equal to 0).

Performing inference on arbitrarily large input images requires that we operate based on image tiles and only on tiles small enough to ft in GPU memory for any single forward pass. To form a tile, the whole image is broken down into non-overlapping regions. This is equivalent to striding across the image with a fixed stride (in pixels). Then, if we include enough local context around each tile to cover the theoretical receptive field of the network, each pixel will have all of the local context it requires. Therefore, while the full image is broken into tiles to perform inference, each pixel individually has all of the information required to be predicted as if the whole image were passed through the network as one block of memory. This work leverages FastImage [24], a high-performance accessor library for processing gigapixel images in a tile-based manner. Figure 1 shows a cropped region from an example (20000 × 20000) pixel stem cell microscope image (left), as well as the segmentation result produced by the model applied to the full image with a correct halo (center), and the segmentation result from tiling without the halo (right).

**Fig. 2:**
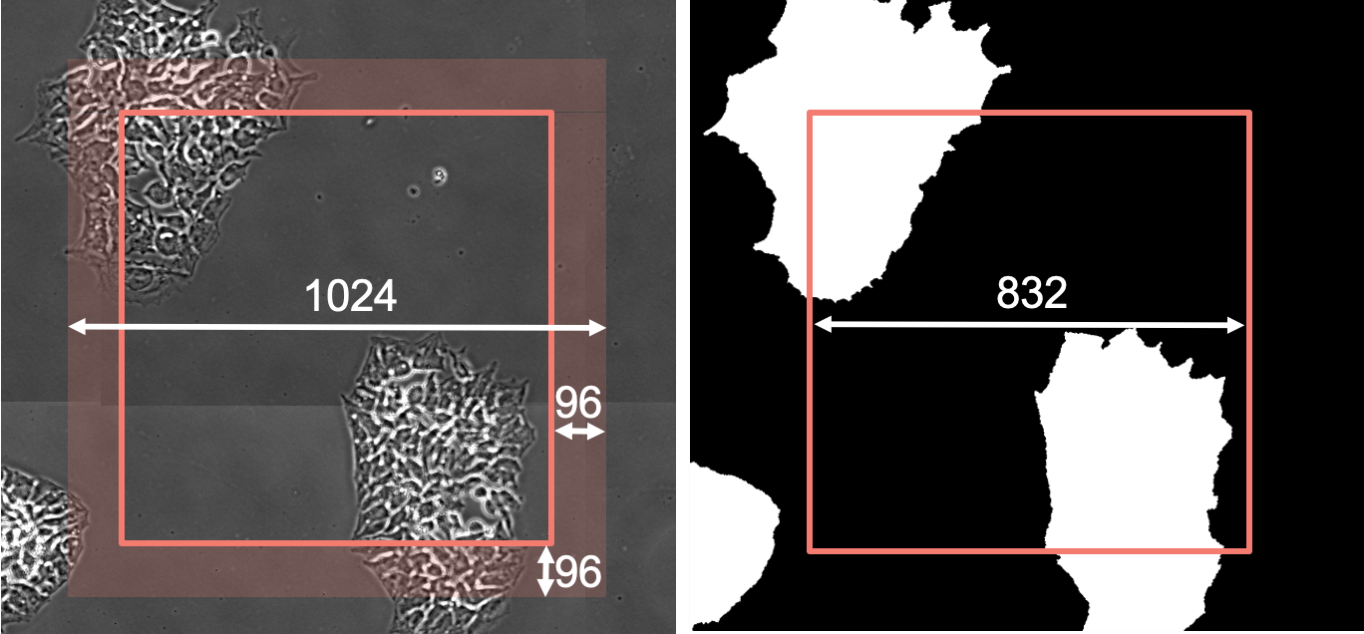
Left: ZoR A (832 pixel ×832 pixel square) with a 96 pixel surrounding halo (shaded area) which combine to make the (1024 × 1024) pixel input tile required to infer the (832 × 832) pixel output tile (ZoR). Right: segmentation output tile showing the ZoR contribution to the final segmented image.

Each dimension of the square input tile is then defined as *inputTileSize* = *ZoR* + 2 × *Halo*. Figure 2 shows an example where a 832 × 832 pixel ZoR is shown as a square with a 96 pixel halo surrounding the ZoR. Since the local context provided by the pixels in the halo is required to correctly compute the output tile, the GPU input is 832 + (2 × 96) = 1024 pixels per spatial dimension.

In other words, the image is broken down into the non-overlapping ZoR (*i.e.*, output tiles). For each ZoR, the local context defined by the halo (where that context is available) is included in the input tile to be passed through the network. For a specific output pixel, if an input pixel is more than half the receptive field away, it cannot influence that output pixel. Therefore, including a halo defined as half the receptive field ensures that pixels on the edge of the ZoR have all the required context.

After passing the input tile through the model, the ZoR within the prediction (without the halo) is copied to the output image being constructed in central processing unit (CPU) memory. Note, the network output is the same size as its input, *ZoR* + (2 × *Halo*). The ZoR needs to be cropped out from the prediction so only those pixels with full context are used to build the final result. The halo provides the network with all the information it needs to make correct, exact predictions for the entirety of the output ZoR. We call this a tiling-error free method because each ZoR is responsible for a specific zone of the output image.

This tile-based inference can be thought of as a series of forward passes, each computing a subregion (ZoR) of the feature maps that would be created while performing inference on the whole image in one pass. In summary, each tile's feature maps are created (forward pass), its ZoR output is extracted, and then the GPU memory is recycled for the next tile. By building each ZoR result in a separate forward pass we can construct the network output within a fixed GPU memory footprint for arbitrarily large images.

## 3.1 U-Net Case Study

Here we use U-Net [2] as a case study example of FCNs. Nonetheless, the presented tiling concept applies to any FCN (without dynamic or variable receptive fields), just the specific numerical values will be different.

Note: for the purpose of brevity we will use 'up-conv' (as the U-Net paper does) to refer to fractionally strided convolutions with a stride of 1*/*2, which doubles the feature map spatial resolution [25].

## 3.1.1 Determining The Halo

The halo must be half the receptive field (*halo* = [*receptive field/*2]). The general principle is to sum the values along the longest path through the network, the product of half the receptive field for each convolutional kernel, and the stride that kernel has across the input image. The stride a specific convolution kernel has across the input image is a combination of that kernel's stride with respect to its feature map and the downsampling factor between the input image size and the spatial size of that feature map. The downsampling factor is determined by the number of spatial altering layers between the input image and a specific layer.

Let U-Net be described by an ordered sequence of convolutional layers *c* = 0, *..., N* - 1 with each layer being associated with a level *l_c_* and a square kernel *k_c_* × *k_c_*. For the network, *N* defines the number of convolutional layers along the longest path from input to output.

Let us define the level *l_c_* of an encoder-decoder network architecture as the number of max-pool^2^2Convolutions with a stride of 2 can also be used to halve the spatial size of the feature maps, but they will affect the receptive field.  layers minus the number of up-conv layers between the input image and the current convolutional layer *c* along the longest path through the network. Levels start at 0; each max pool encountered along the longest path increases the level by 1 and each up-conv reduces the level by 1.

## 3.1.2 General Halo Calculation

The required halo can be calculated according to Eq. 1 for a UNet-type FCN architecture. This equation can be considered to be a special case of the more general framework presented by Araujo *et al.* [7] for computing CNN receptive fields; however, it enables an exploration of how each UNet element contributes to the receptive field.

$Halo=\overset{N-1}{\underset{c=0}{\Sigma }}2^{lc}\left[\frac{kc}{2}\right]$(1)

The halo is a sum over every convolutional layer index *c* from 0 to *N* - 1 encountered along the longest path from the input image to the output image. Equation 1 has two terms. The $2^{lc}$ term is the number of pixels at the input image resolution that correspond to a single pixel within a feature map at level *l_c_*. Therefore, at level *l_c_* = 4, each pixel in the feature map equates to 2^4^ = 16 pixels at the input image resolution. This $2^{lc}$ term is multiplied by the second term $\frac{k_{c}}{2}$which determines, for a given *c*, the number of pixels of local context that are required at that feature map resolution to perform the convolution.

### 3.1.3 U-Net Configuration

We have made two modifications to the published U-Net architecture.

1. Normalization: Batch normalization [26] was added after the activation function of each convolutional layer because it is current good practice in the CNN modeling community.
2. Convolution Type: The convolutional padding scheme was changed to SAME from VALID as used in the original paper [2].

Batch normalization will not prevent numerically identical results compared to performing inference on the whole image in a single pass if correct inference procedures are followed, where the normalization statistics are frozen, having been estimated during the training process.

The original U-Net paper used VALID type convolutions^3^3For an excellent review of convolutional arithmetic, including transposed convolutions (i.e., up-conv), see "A guide to convolutional arithmetic for deep learning by Dumoulin and Visin" [25]. which shrink the spatial size of the feature maps by 2 pixels for each layer [25]. Switching to SAME type convolutions preserves feature map size. See Appendix D for additional explanation.

There is one additional constraint on U-Net that needs to be mentioned. Given the skip connections between the encoder and decoder elements for matching feature maps, we need to ensure that the tensors being concatenated together are the same size. This can be restated as requiring the input size to be divisible by the largest stride across the input image by any kernel. For U-Net this stride is 16 pixels (derivation in Appendix D). Another approach to ensuring consistent size between the encoder and decoder is to pad each up-conv layer to make its output larger and then crop it to the target feature map size. That is a less elegant solution than enforcing a tile size constraint and potentially padding the input image.

### 3.1.4 Halo Calculation for U-Net

The published U-Net (Figure 1 from [2]) has one level per horizontal stripe of layers. The input image enters on level *l_c_*_=0_ = *l_c_*_=1_ = 0. The first max-pool layer halves the spatial resolution of the network, changing the level. Convolution layers *c* = {2, 3} after that first max-pool layer up to the next max-pool layer belong to level *l_c_*_=2_ = *l_c_*_=3_ = 1. This continues through level 4, where the bottleneck of the U-Net model occurs. In U-Net's Figure 1 [2], the bottleneck is the feature map at the bottom which occurs right before the first up-conv layer. After the bottleneck, the level number decreases with each subsequent up-conv layer, until level *l_N_*_-1_ = 0 right before the output image is generated.

The halo computation in Eq. 1 can be simplified for U-Net as shown in Appendix A. Appendix B shows a numerical example for computing the halo size using Eq. 1.

Following Eq. 1 for U-Net results in a minimum required halo of 92 pixels in order to provide the network with all of the local context it needs to predict the outputs correctly. This halo needs to be provided both before and after each spatial dimension, and hence the input image to the network will need to be 2 × 92 = 184 pixels larger. This value is exactly the number of pixels by which the original U-Net paper shrunk the output to avoid using SAME convolutions; a 572 pixel input shrunk by 184 results in the 388 pixel output [2]. However, this runs afoul of our additional restriction on the U-Net input size, which requires images to be a multiple of 16. So rounding up to the nearest multiple of 16 results in a halo of 96 pixels. Since one cannot simply adjust the ZoR size to ensure (*ZoR* + *Halo*)%16 = 0 due to convolutional arithmetic, we must explore constraints on image partitioning.

## 3.2 Constraints on Image Partitioning

Our tile-based processing methodology operates on the principle of constructing the intermediate feature map representations within U-Net in a tile-based fashion, such that they are numerically identical to the whole image being passed through the network in a single pass. Restated another way, the goal is to construct an input image partitioning scheme such that the ZoR constructs a spatial subregion of the feature maps that would exist if the whole image were passed through the network in a single pass.

## 3.2.1 Stride Selection

**Fig. 3:**
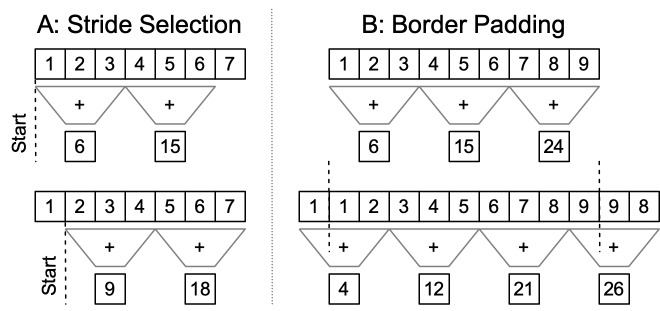
(Left): Simplified one-dimensional example of an addition kernel of size 3 being applied at an offset less than the kernel size, producing different results (compare top and bottom sums of 1, 2, and 3 or 2, 3, and 4). (Right): Simplified 1D example of refection padding (reflected through dotted line), causing a different stride pattern across a set of pixels. The altered stride prevents the tile-based processing from collaboratively building subregions of a single feature map.

To properly construct this feature map subregion, the tiling cannot stride across the input image in a different manner than would be used to perform inference on the whole image. The smallest feature map in U-Net is spatially 16× smaller than the input image. Therefore, 16 pixels is the smallest offset one can have between two tile-based forward network passes while having both collaboratively build subregions of a single feature map representation. Figure 3 shows a simplified one-dimensional example with a kernel of size 3 performing addition. When two applications of the same kernel are offset by less than the size of the kernel, they can produce different results. For U-Net, each (16 × 16) pixel block in the input image becomes a single pixel in the lowest spatial resolution feature map. A stride other than a multiple of 16 would result in subtly different feature maps because each feature map pixel was constructed from a different set of (16 × 16) input pixels.

This requirement means that the tiling of the full image always needs to start at the top-left corner and stride across in a multiple of 16. However, this does not directly answer the question as to why we cannot have a non-multiple of 16 halo value.

## 3.2.2 Border Padding

The limitation on the halo comes from the fact that if we have arbitrary halo values, we will need to use different padding schemes between the full image inference and the tile-based inference to handle the image edge effects. Figure 3 shows for a 1D case how refection padding can (1) alter the stride across the full image, which needs to be maintained as a multiple of 16 to collaboratively build subregions of a single feature map, and (2) change the refection padding required to have an input image for which the spatial dimensions are a multiple of 16. Refection padding is preferred since it preserves image statistics locally.

## 3.2.3 ZoR and Halo Constraints

Both problems, (1) collaboratively building feature maps and (2) different full image edge refection padding requirements disappear if both the ZoR and the halo are multiples of 16. Thus, we constrain the final values of ZoR and halo to be the closest higher multiple of the ratio *F* between the image size *I* and minimum feature map size (Eq. 2), where *F* = 16 for the published U-Net:

$F=\frac{\min\left\{H_{I},W_{I}\right\}}{\min_{\forall l_{c}}\left\{H_{l_{c}},W_{l_{c}}\right\}}$

$Halo*=F\left\lceil \frac{Halo}{F}\right\rceil$

$ZoR=F\left\lceil \frac{ZoR}{F}\right\rceil$ (2)

where *H_I_* and *W_I_* are the input image height and width dimensions, respectively, Halo^*^ is the adjusted halo value to accommodate stride constraints, and *H_l__c_* and *W_l__c_* are the feature map height and width dimensions, respectively.

## 4 Experimental Results

### 4.1 Data Set

We used a publicly accessible data set acquired in phase contrast imaging modality and published by Bhadriraju *et al.* [27]. The data set consists of three collections, each with around 161 time-lapse images at roughly (20000 × 20000) pixels per stitched image frame with 2 bytes per pixel.

### 4.2 Exact Tile-Based Inference Scheme

Whether performing inference on the whole image in a single forward pass or using tile-based processing, the input image size needs to be a multiple of 16 as previously discussed. Refection padding is applied to the input image to enforce this size constraint before the image is decomposed into tiles.

Let us assume that we know how big an image we can ft into GPU memory, for example, (1024 × 1024) pixels. Additionally, given that we are using U-Net, we know that the required halo is 96 pixels. In this case, the zone of responsibility is *ZoR* = 1024 - (2 × *Halo*) = 832 pixels per spatial dimension. Despite performing inference on (1024 × 1024) pixel tiles on the GPU per forward pass, the stride across the input image is 832 pixels because we need non-overlapping ZoR. The edges of the full image do not require halo context to ensure identical results when compared with a single inference pass. Intuitively, the true context is unknown, since it is outside the existing image.

In the last row and column of tiles, there might not be enough pixels to fill out a full (1024 × 1024) pixel tile. However, because U-Net can alter its spatial size on demand, as long as the tile is a multiple of 16, a narrower (last column) or shorter (last row) tile can be used.

### 4.3 Errors due to an Undersized Halo

To experimentally confirm that our out-of-core image inference methodology does not impact the results we determined the largest image we could process on our GPU, performed the forward pass, and saved the resulting softmax output values as ground truth data. We then processed the same image using our tiling scheme with varying halo values. We show that there are numerical differences (greater than floating point error) when using halo values less than 96.

Our U-Net model was trained to perform binary (foreground/background) segmentation of the phase contrast microscopy images. The largest image on which we could perform inference given our GPU with 24 GB of memory was (3584 × 3584) pixels. Therefore, we created 20 reference inference results by cropping out *K* = 20 random (3584 × 3584) subregions of the data set. Tile-based out-of-core inference was performed for each of the 20 reference images using a tile size of 512 pixels (thereby meeting the multiple of 16 constraint) with halo values from 0 to 96 pixels in 16 pixel increments.

The tiling codebase seamlessly constructs the output in CPU memory as if the whole image had been inferred in a single forward pass. So our evaluation methodology consists of looking for differences in the output softmax values produced by the reference forward pass (*R*) as well as the tile-based forward pass (*T*).

We used the following two metrics for evaluation: root mean squared error (RMSE) of the softmax outputs as given in Eq. 3 and misclassification error (ME) of the resulting binary segmentation masks as given in Eq. 4. We also included misclassification error rate (MER) as in Eq. 5 where the ME is normalized by the number of pixels; and relative runtime, where the computation time required to perform inference is shown relative to the runtime without the tiling scheme. This runtime highlights the tradeoff to be made between the error introduced due to the out-of-core GPU inference and the computational overhead required to do so. The MER metric can be multiplied by 100 to compute the percent of pixels with errors due to the tiling scheme. All metrics were averaged across the *K* = 20 reference images.

$RMSE=\frac{1}{K}\sum_{i=1}^{K}\sqrt{\left(\frac{{\sum_{i=1}^{m}\sum_{j=1}^{n}\left(Rij-Tij\right)}^{2}}{mn}\right.}$ (3)

$ME=\frac{1}{K}\sum_{i=1}^{m}\left.\sum_{i=1}^{m}\sum_{j=1}^{n}\left[Rij\neq Tij\right]\right)\left(\right.$ (4)

$MER=\frac{1}{K}\sum_{i=1}^{K}\left(\frac{\sum_{i=1}^{m}\sum_{j=1}^{m}\left[Rij\neq Tij\right]}{nm}\right)$ (5)

The total inference error is a composite of model error (which is directly minimized by gradient descent during training) and tiling error. We demonstrate a zero error contribution from tiling in the case of trained and untrained U-Net models (or minimum and maximum inference errors due to a model).

**Table 1:** Error Metrics for Tile Size = 512

TileSize	ZoR	Halo	RMSE	ME	*σ* (ME)	MER	RelativeRuntime
3584	n/a	n/a	0.0	0.0	0.0	0.0	1.0
512	512	0	1.11 × 10^-2^	7773.4	1350	6.1 × 10^-4^	1.08
512	480	16	6.35 × 10^-3^	5455.4	1420	4.2 × 10^-4^	1.31
512	448	32	3.29 × 10^-3^	2372.2	670	1.8 × 10^-4^	1.36
512	416	48	1.95 × 10^-3^	1193.7	350	9.3 × 10^-5^	1.61
512	384	64	7.79 × 10^-4^	434.1	141	3.4 × 10^-5^	1.85
512	352	80	1.50 × 10^-4^	71.6	27	5.6 × 10^-6^	2.21
512	320	96	4.17 × 10^-10^	0.0	0.0	0.0	2.58

For the trained model, the error metrics are shown in Table 1 with 512 pixel tiles ^4^4All results were generated on an Intel Xeon 4114 CPU with an NVIDIA Titan RTX GPU using Python 3.6 and TensorFlow 2.1.0 running on Ubuntu 18.04.. Once the required 96 pixel halo is met, the RMSE falls into the range of floating point error, and the ME goes to zero. Beyond the minimum required halo, all error metrics remain equivalent to the minimum halo. The first row shows the data for the whole image being inferred without the tiling scheme. The ME metric is especially informative, because when it is zero, the output segmentation results are identical regardless of whether the whole image was inferred in a single pass or it was decomposed into tiles. Table 1 highlights the engineering tradeoff that must be made, where obtaining zero inference error requires 2.58× the wall clock runtime. The MER with naive tailing is 6.1 × 10^-4^ or 0.06%. Depending on your application, this level of error might be acceptable despite the potential for edge effects between the non-overlapping tiles. One consideration is that larger tile sizes are more computationally efficient because the ratio of the ZoR area to the tile area increases.

For the untrained model, results are shown in Table 3 in Appendix C with 1024 pixel tiles. The results were generated using an untrained 4 class U-Net model, in which weights were left randomly initialized. Additionally, the image data for that result was normally distributed random noise with *μ* = 0, *σ* = 1. The error coming from tile-based processing was zero once the required halo was met.

### 4.4 Errors due to Violation of Partitioning Constraints

**Fig. 4:**
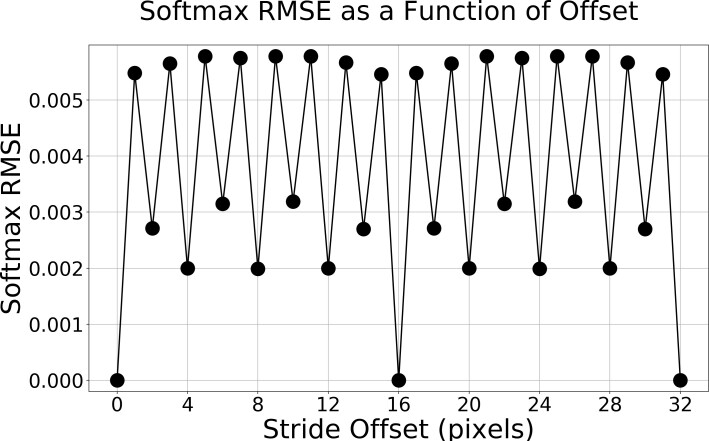
Impact of the stride offset on the RMSE of the U-Net softmax output.

To demonstrate how the inference results differ as a function of how the network strides across the input image, we have constructed 32 overlapping (2048 × 2048) pixel subregions of an image; each offset from the previous subregion start by 1 pixel. So the first subregion is [*x_st_, y_st_, x_end_, y_end_*] = [0, 0, 2048, 2048], while the second subregion is [1, 0, 2049, 2048], and so on. In order to compare the inference results without any edge effects confounding the results, we only computed the RMSE (Eq. 3) of the softmax output within the area in common between all 32 images, inset by 96 pixels; [128, 96, 1920, 1952]. The results are shown in Figure 4, where identical softmax outputs only happen when the offset is a multiple of 16.

### 4.5 Application to a Fully Convolutional DenseNet

Up to this point, we have shown that our ZoR and halo tiling scheme produces error-free out-of-core semantic segmentation inference for arbitrarily large images when using the published U-Net architecture [2]. This section demonstrates the tiling scheme on a fully convolutional DenseNet configured for semantic segmentation [28]. DenseNets [29] replace stacked convolutional layers with densely connected blocks, where each convolutional layer is connected to all previous convolutional layers in that block. Jegou *et al.* [28] extended this original DenseNet idea to create a fully convolutional DenseNet based semantic segmentation architecture.

While the architecture of DenseNets significantly differs from U-Net, the model is still fully convolutional and thus our tiling scheme is applicable. Following Eq. 1 for a FC-DenseNet-56 [28] model produces a required halo value of 377. This is significantly higher than U-Net due to the architecture depth. FC-DenseNet-56 also has a ratio between the input image size and the smallest feature map of *F* = 32. Therefore, the inference image sizes need to be a multiple of 32, not 16 like the original U-Net. Thus, the computed 377 pixel halo is adjusted up to 384.

The error metrics for FC-DenseNet-56 as a function of halo are shown in Table 4 in Appendix C. This numerical analysis relies on versions of the same 20 test images from the U-Net analysis, but they are cropped to (2304 × 2304), which was the largest image on which we were able to perform inference using FC-DenseNet-56 on our 24 GB GPU in a single forward pass.

### 4.6 Halo Approximation via Effective Receptive Field

The halo value required to achieve error free inference increases with the depth of the network. For example, see Table 2 which shows the theoretical halo values (computed using Eq. 1) for a few common semantic segmentation architectures. The deeper networks, like FC-DenseNet-103, require very large halo values to guarantee error-free tile-based inference.

Using the empirical effective receptive field estimation method outlined by Luo *et al.* [19], which consists of setting the loss to 1 in the center of the image and then back propagating to the input image, we can automatically estimate the required halo. This method produces an estimated halo of 96 pixels for our trained U-Net [2], which is exactly the theoretical halo. This matches the data in Tables 1 and 3, where the ME metric did not fall to zero until the theoretical halo was reached. On the other hand, according to Table 4 for FC-DenseNet-56, there is no benefit to using a halo larger than 192 despite the theoretical required halo (receptive field) being much larger. This is supported by the effective receptive field estimated halo of 160

**Table 2:** Theoretical Radii for Common Segmentation Architectures

Architecture	Halo (pixels)
U-Net [2]	96
SegNet [1]	192
FCN-VGG16 [3]	202
FC-DenseNet-56 [28]	384
FC-DenseNet-67 [28]	480
FC-DenseNet-103 [28]	1120

pixels; which is just below the empirically discovered minimum halo of 192. Using the effective receptive field for estimating the required halo is not foolproof, but it provides a good proxy for automatically reducing the tiling error. The effective receptive field will always be less than or equal to the true network's potential receptive field because convolution kernel weights can learn to ignore information, but they cannot increase the kernel size.

## 5 Conclusions

This paper outlined a methodology for performing error-free segmentation inference for arbitrarily large images. We documented the formulas for determining the tile-based inference scheme parameters. We then demonstrated that the inference results are identical regardless of whether or not tiling was used. These inference scheme parameters were related back to the theoretical and effective receptive fields of deep convolutional networks as previously studied in literature [19]. The empirical effective receptive field estimation methods of Luo *et al.* [19] were used to provide a rough estimate of the inference tiling scheme parameters without requiring any knowledge of the architecture. While we used U-Net and FC-DenseNets as example FCN models, these principles apply to any FCN model while being robust across different choices of tile size.

In this work we did not consider any FCN networks with dilated convolutions, which are known to increase the receptive field side of the network. We will include this extension in future work as well as tradeoff evaluations of the relationships among relative runtime, GPU memory size, and maximum tile size.

## 6 Test Data and Source Code

The test data and the Tensorfow v2.x source code are available from public URLs^5^5https://isg.nist.gov/deepzoomweb/data/stemcellpluripotency; https://github.com/usnistgov/semantic-segmentation-unet/tree/ooc-inference.. While the available codebase in theory supports arbitrarily large images, we made the choice at implementation time to load the whole image into memory before processing it through the network. In practice, this means the codebase is limited to performing inference on images that ft into CPU memory. However, a file format that supports reading sub-sections of the whole image would support inference of disk-backed images which do not ft into CPU memory.

## A Appendix A: Derivation of the Simplified Halo Formula

Let us assume that in the entire U-Net architecture the kernel size is constant *k_c_* = *k* = *const* and each level has the same number of convolutional layers on both decoder and encoder sides *n_l_* = *n* = *const*. If these constraints are satisfied, then the general formula for determining the halo can be simplified as follows:

$\begin{array}{lcr} & \begin{array}{r} Halo=\sum_{c=1}^{N}2^{l_{c}}\lfloor \frac{k_{c}}{2}\rfloor \\ =\lfloor \frac{k}{2}\rfloor \times \sum_{c=1}^{N}2^{l_{c}}\\ =\lfloor \frac{k}{2}\rfloor \times (2\times \sum_{m=0}^{M-1}(2^{m}\times n)+2^{M}\times n)\\ =\lfloor \frac{k}{2}\rfloor \times n\times (2\times \frac{1\times (1-2^{M})}{1-2})+2^{M})\\ =\lfloor \frac{k}{2}\rfloor \times n\times (3\times 2^{M}-2) \end{array} & \end{array}$ (6)

where *M* is the maximum U-Net level *M* = max_∀_*_c_*{*l_c_*} .

For U-Net architecture, the parameters are *k* = 3, *n* = 2 and *M* = 4, and the equation yields *Halo* = 92.

For DenseNet architecture, the parameters are *k* = 3, *n* = 4 and *M* = 5, and the equation yields *Halo* = 376. This value differs by one from the value computed according to Eq. 6 because the DenseNet has asymmetry between the first encoder layer with a kernel size *k* = 3 and the last decoder layer with a kernel size *k* = 1.

## B Appendix B: Example U-Net Halo Calculation

Following Eq. 1 for U-Net results in a required halo of 92 pixels in order to provide the network with all of the local context it needs to predict the outputs correctly. With *k* = 3, $\frac{k_{c}}{2}$reduces to $\frac{3}{2}$= 1. The halo computation for U-Net thus reduces to a sum of 2*^l^^c^* terms for each convolutional layer encountered along the longest path from input to output as shown in Eq. 7.

$Halo=\sum_{c=0}^{17}2^{l_{c}}$ (7)

By substituting the level numbers for each convolutional layer from 0 to 17 as shown in Eq. 8, one obtains the minimum halo value of 92 pixels.

*l_c_* = {0, 0, 1, 1, 2, 2, 3, 3, 4, 4, 3, 3, 2, 2, 1, 1, 0, 0} _(8)_

$92=2^{0}+2^{0}+2^{1}+2^{1}+2^{2}+2^{2}+2^{3}+...$

Similarly, according to Eq. 6, the calculation simplifies to:

$\begin{array}{lcr} & \begin{array}{r} M=\max_{\forall c}\;l_{c}=4\\ k_{c}=k=1\\ n_{l}=n=2\\ 92=1\times 2\times (3\times 2^{4}-2) \end{array} & \end{array}$ (9)

## C Appendix C: Error Metrics

**Table 3:** UnTrained U-Net Error Metrics for Tile Size = 1024

TileSize	ZoR	Halo	RMSE	ME	NME	RelativeRuntime
3584	n/a	n/a	0.0	0.0	0.0	1.0
1024	1024	0	2.36 × 10^-4^	21856.9	1.7 × 10^-3^	1.08
1024	992	16	1.05 × 10^-6^	234.8	1.8 × 10^-5^	1.23
1024	960	32	1.92 × 10^-7^	49.7	3.9 × 10^-6^	1.30
1024	928	48	5.47 × 10^-8^	13.2	1.0 × 10^-6^	1.35
1024	896	64	1.44 × 10^-8^	2.8	2.1 × 10^-7^	1.31
1024	864	80	5.87 × 10^-9^	1.4	1.1 × 10^-7^	1.5
1024	832	96	3.54 × 10^-10^	0.0	0.0	1.58

**Table 4:** Error Metrics for FC-DenseNet Tile Size = 1152

TileSize	ZoR	Halo	RMSE	ME	NME	RelativeRuntime
2304	n/a	n/a	0.0	0.0	0.0	1.0
1152	1152	0	5.40 × 10^-3^	821.5	1.5 × 10^-4^	1.15
1152	1088	32	1.81 × 10^-3^	376.1	7.1 × 10^-5^	1.42
1152	1024	64	9.16 × 10^-4^	168.0	3.2 × 10^-5^	1.54
1152	960	96	3.36 × 10^-4^	51.6	9.7 × 10^-6^	1.59
1152	896	128	5.63 × 10^-5^	5.3	1.0 × 10^-6^	1.67
1152	832	160	4.97 × 10^-6^	0.2	4.7 × 10^-8^	1.76
1152	768	192	2.44 × 10^-7^	0.0	0.0	2.32
1152	704	224	1.92 × 10^-8^	0.0	0.0	2.22
1152	640	256	1.37 × 10^-8^	0.0	0.0	2.33
1152	576	288	1.44 × 10^-8^	0.0	0.0	2.39
1152	512	320	1.37 × 10^-8^	0.0	0.0	2.52
1152	448	352	1.45 × 10^-8^	0.0	0.0	4.65
1152	384	384	1.37 × 10^-8^	0.0	0.0	5.89

## D Appendix D: Convolution Padding

The original U-Net paper used VALID type convolutions which shrink the spatial size of the feature maps by 2 pixels for each layer [25]. Figure 2.1 from Dumoulin and Visin [25] shows an illustration showing why VALID causes the feature maps to shrink. The effect of VALID convolutions can also be seen in the frst layer of the original U-Net, where the input image size of (572 × 572) pixels shrinks to (570 × 570) pixels [2]. SAME type convolutions requires zero padding to be applied to each feature map within each convolutional layer ensure the output has the same spatial size as the input. Figure 2.3 from [25] shows an illustration where a input (5 × 5) remains (5 × 5) pixels after the convolution is applied. While VALID type convolutions avoid the negative effects of the zero padding within SAME type convolutions, which can affect the results as outlined by Huang et al. [15], it is conceptually simpler to have input and output images of the same size. Additionally, our tiling scheme overcomes all negative effects that zero padding can introduce, justifying the choice of SAME type convolutions.

The change to SAME type convolutions introduces an additional constraint on U-Net that needs to be mentioned. Given the skip connections between the encoder and decoder elements for matching feature maps, we need to ensure that the tensors being concatenated together are the same size. This can be restated as requiring the input size to be divisible by the largest stride across the input image by any kernel. Another approach to ensuring consistent size between the encoder and decoder is to pad each up-conv layer to make its output larger and then crop to the target feature map size. We feel that is a less elegant solution than enforcing a tile size constraint and potentially padding the input image.

The feature map at the bottleneck of U-Net is spatially 16× smaller than the input image. Therefore, the input to U-Net needs to be divisible by 16. As we go deeper into a network we trade spatial resolution for feature depth. Given a (512 × 512) pixel input image, the bottleneck shape will be *N* × 1024 × 32 × 32 (assuming NCHW^6^ 6 NCHW Tensor dimension ordering: N (batch size), Channels, Height, Width dimension ordering with unknown batch size). Thus the input image height divided by the bottleneck feature map height is ^$\frac{512}{32}$^= 16. However, if the input image is (500 × 500) pixels, the bottleneck would be (in theory) *N* × 1024 × 31.25 × 31.25. When there are not enough input pixels in a feature map to perform the 2 × 2 max pooling, the output feature map size is the floor of the input size divided by 2. Thus, for an input image of (500 × 500) pixels the feature map heights after each max pooling layer in the encoder are: [500, 250, 125, 62, 31]. Now following the up-conv layers through the decoder, each of which doubles the spatial resolution, we end up with the following feature map heights: [31, 62, 124, 248, 496]. This results in a different feature map spatial size at the third level; encoder 125, decoder 124. If the input image size is a multiple of 16, then this mismatch cannot happen.
